# 24-Month assessment of respiratory function in patients hospitalized for severe SARS-CoV-2 pneumonia: a follow-up study

**DOI:** 10.1007/s11739-025-04153-5

**Published:** 2025-11-03

**Authors:** Laura Pini, Michele Guerini, Jordan Giordani, Guido Levi, Nicola Latronico, Simone Piva, Elena Peli, Roberto Benoni, Alessandro Pini, Giovanni Zucchi, Stefano Piras, Yehia El Masri, Dina Visca, Marco Caminati, Gianenrico Senna, Carolina De Ciuceis, Claudia Agabiti Rosei, Maria Lorenza Muiesan, Claudio Tantucci

**Affiliations:** 1 Department of Clinical and Experimental Sciences, University of Brescia & ASST - Spedali Civili di Brescia, Brescia, Italy; 2 Pulmonology Department, ASST – Spedali Civili di Brescia, Brescia, Italy; 3 Department of Anesthesia, Critical Care and Emergency, ASST Spedali Civili University Hospital, Brescia, Italy; 4 Department of Medical and Surgical Specialties, Radiological Sciences and Public Health, University of Brescia, Brescia, Italy; 5 Department of Diagnostics and Public Health, University of Verona, Verona, Italy; 6 Department of Emergency, Anaesthesiological and Resuscitation Sciences, University Cattolica Sacro Cuore, Rome, Italy; 7 Department of Medicine and Surgery, University of Insubria, Varese, Italy; 8 Department of Medicine and Cardiopulmonary Rehabilitation, Istituti Clinici Scientifici Maugeri IRCCS, Tradate, Italy; 9 Department of Medicine, University of Verona, Verona, Italy; 10 Allergy Unit and Asthma Center, Verona Integrated University Hospital, Verona, Italy; 11 Internal Medicine Unit, ASST Spedali Civili di Brescia, Brescia, Italy

**Keywords:** SARS-CoV-2, Pulmonary function test, DLCO, Lung volumes, Restrictive defect

## Abstract

Long COVID affects multiple body systems, with the respiratory system being particularly vulnerable. This study aimed to analyze the lung ventilatory function and diffusion capacity of patients with severe SARS-CoV-2 pneumonia during a 24-month follow-up course. Ventilatory function and lung diffusion capacity were assessed 6, 12, 18, and 24 months after hospital discharge. Ventilatory parameters, Diffusion Lung Carbon Monoxide (DLCO), and KCO (Carbon Monoxide transfer coefficient) normalization were defined as achieving values > 80% predicted. A total of 222 patients admitted to the Intensive Care Unit (ICU) at ASST Spedali Civili di Brescia, Brescia, Italy, were enrolled. Among the 172 patients who completed the study, 140 (63%) achieved normalization of ventilatory parameters, DLCO, and KCO. The median time to recovery was 4.5 months, and the hazard ratio (HR) decreased by 2% for each year of age increase. The median time to normalize ventilatory parameters (VC, FVC, FEV_1_, FEV1/FVC, TLC, and KCO) was 1.5 months, while the median time to alveolar volume (VA) normalization was 4.5 months. Male gender reduces the odds of normalization for FEV1/FVC and VA. The median time to DLCO normalization was 9 months, with HR reduced by 3.1% as each year of age increased and augmented by 226% in obese subjects. 24 months after severe COVID pneumonia, 14% of patients had persistent ventilatory and/or diffusive defects. Our study documented that male sex, age, and obesity impact the odds of normalization of ventilatory function and diffusive capacity. These findings underline the chronic nature of lung damage following severe COVID-19 pneumonia and the need for long-term follow-ups.

**Supplementary Information:**

The online version contains supplementary material available at 10.1007/s11739-025-04153-5.

## Introduction

Long COVID, also known as post-acute sequelae of SARS-CoV-2 infection (PASC), emerged as a significant health concern following the global COVID-19 pandemic. This condition is characterized by persistent symptoms or new-onset complications continuing for weeks or months after the initial acute phase of COVID-19 infection. While the full spectrum of long-term effects is still being investigated, current evidence suggests that COVID-19 has lasting impacts on various body symptoms, with the respiratory system particularly vulnerable. Respiratory symptoms of Long COVID vary widely, from mild to severe. Common symptoms include persistent cough, shortness of breath, chest pain, and reduced exercise tolerance. These symptoms can significantly impact daily activities and quality of life, leading to increased healthcare utilization and economic burden [1–3].

Pathophysiological mechanisms underlying the respiratory effects of Long COVID are complex and multifaceted. One proposed mechanism involves direct viral-induced lung damage, resulting in fibrosis and reduced lung function. Additionally, the hyperinflammatory state associated with severe COVID-19 may lead to persistent inflammation and immune dysregulation, which can contribute to ongoing respiratory symptoms. Vascular complications, including microthrombus formation and endothelial dysfunction, may also contribute to the long-term respiratory sequelae of COVID-19. Imaging studies revealed persistent abnormalities in the lungs of Long COVID patients, even in those who experienced mild acute illness. These abnormalities include ground-glass opacities, consolidations, and fibrotic changes. Pulmonary function tests often reveal reduced lung diffusion capacity for carbon monoxide (DLCO) and restrictive ventilatory patterns, resulting in impaired gas exchange and decreased lung volumes [4–9].

The long-term prognosis for patients with respiratory manifestations of Long COVID remains uncertain. While some individuals experience gradual improvement over time, others struggle with persistent symptoms for extended periods. This outcome variability highlights the need for personalized patient care and long-term follow-up approaches. Management strategies for respiratory symptoms in Long COVID are evolving as our understanding of the condition grows. Current approaches often include pulmonary rehabilitation programs, breathing exercises, and symptomatic management. The implications of Long COVID for public health are significant. Long-term respiratory complications following COVID-19 infection underline the importance of prevention strategies, including vaccination and public health measures to reduce transmission [10, 11].

Additionally, healthcare systems must prepare for the ongoing care needs of Long COVID patients, which may consume resources and require specialized multidisciplinary teams. Research efforts continue to improve our understanding of the natural history of Long COVID, identify risk factors for persistent symptoms, and develop targeted interventions. Large-scale longitudinal studies are crucial for elucidating the long-term trajectory of respiratory function in affected individuals and for tailoring evidence-based treatments [12, 13].

This paper aims to analyze the long-term effects of SARS-CoV-2 pneumonia on ventilatory function and lung diffusion capacity at different time points during a 24-month follow-up course. In particular, the primary aim of this study was to evaluate the long-term recovery of ventilatory function and lung diffusion capacity in patients who survived severe SARS-CoV-2 pneumonia over a 24-month follow-up period. We hypothesized that demographic factors such as age, sex, and obesity influence the timing and likelihood of normalization of lung function parameters, including VC, FVC, FEV1, TLC, DLCO, KCO, and alveolar volume.

## Materials and methods

### Subjects and measurements

Ventilatory function and diffusion capacity of the lung of a selected population admitted to the Intensive Care Unit (ICU) of the ASST—Spedali Civili di Brescia, Brescia, Italy, and survived severe SARS-CoV-2 pneumonia have been analyzed at 6, 12, 18, and 24 months after hospital discharge. Patients were enrolled consecutively and had to meet the following criteria: age over 18, a laboratory-confirmed SARS-CoV-2 infection documented through real-time reverse transcription-polymerase chain reaction (RT-PCR), pulmonary involvement diagnosed with clinical evaluation and chest X-ray or HRCT imaging, and critical disease state with the need for noninvasive (NIV) and/or orotracheal intubation (IOT) mechanical ventilation. Patients who could not perform spirometry and those with a history of known obstructive, restrictive, or mixed ventilatory defects caused by previous respiratory diseases were excluded from the study.

A physical examination and complete pulmonary function test with maximal flow-volume curve, lung volumes, and Lung Diffusion Capacity for Carbon Monoxide (DLCO) measurements were performed for each patient. Recorded parameters included slow and forced vital capacity (VC and FVC), forced expiratory volume at the first second of maximal expiration (FEV1), and the FEV1/VC% ratio. Lung volumes were measured through the inert gas dilution technique using the Helium closed-circuit multi-breaths method, including functional residual capacity (FRC), residual volume (RV), and total lung capacity (TLC), while DLCO, along with alveolar volume (VA) and coefficient transfer for CO (KCO) through single breath technique (BIOMEDIN Instruments, Padua, Italy). DLCO and KCO have been adjusted for patients’ hemoglobin levels. Subjects were considered to have reached a normalization of lung function if they achieved an FEV1/VC% % ratio above the Lower Limit of Normal (LLN), a total lung capacity (TLC) > 80% predicted, and DLCO > 80% predicted during follow-up. Once these criteria were met, patients were no longer followed. In the presence of FEV1/VC% % ratio > LLN, values of Total Lung Capacity (TLC) < 80% pred. and DLCO < 80% pred. were chosen to identify the presence of restrictive ventilatory defect and/or lung diffusion capacity reduction, respectively, and were followed until normalization or otherwise until the follow-up end at 24 months. Patients who stopped attending visits were considered lost to follow-up.

Data were described according to the pulmonary function test results, and a dedicated database was designed to report demographic data, BMI, and the relative percentages of the predicted values for respiratory function parameters, including TLC, alveolar volume (VA), KCO, and DLCO. DLCO adjustments based on hemoglobin levels were performed using the equations described by J. M. B. Hughes and N. B. Pride and recommended by the ERS [14, 15]:

$$\mathrm{DLCOcorr}=\mathrm{DLCO}\frac{\mathrm{obs}\times 10.2+\left[\mathrm{Hb}\right]}{1.7\times \left[\mathrm{Hb}\right]} \text{for adult males}$$

$$\mathrm{DLCOcorr}=\mathrm{DLCO}\frac{\mathrm{obs}\times 9.38+\left[\mathrm{Hb}\right]}{1.7\times \left[\mathrm{Hb}\right]}\text{for adult females}.$$

This work was carried out in accordance with the Declaration of Helsinki and is part of the project “Long-term Follow-up in Survivors of Critical Illness” registered on ClinicalTrials.gov—ID: NCT04608994 and approved by the Brescia Ethics Committee (NP 2595).

### Statistics

Data were expressed as mean ± standard deviation (SD), and categorical variables were recorded as frequencies and percentages. Where applicable, demographic data of the recruited population were analyzed using the Student’s *t*-test for continuous variables and the Fisher’s exact test for categorical variables.

The Kaplan–Meier model examined the median time to normalization of respiratory function. The association between the normalization of spirometry parameters and demographic characteristics of the recruited population was studied by semi-parametric Cox regression analysis. This model estimates the effect of independent variables on the risk of an event at a given time using a time-based risk function. The Hazard Ratio (HR) derived from this analysis is an index that measures the effect of predictor variables on the risk of an event. An HR > 1 indicates that an increase in the predictor variable is associated with an increased risk of the event. An HR < 1 indicates that an increase in the predictor variable is associated with a decrease in the risk of the event. An HR = 1 indicates that the predictor variable does not affect the risk of the event.

Survival analysis was performed using fixed timepoint data, where hospitalization is the starting point (t0), the last follow-up corresponds to the right limit of the interval (tR), and the previous follow-up point corresponds to the left limit of the interval (tL). Statistical significance was assessed for *p*-values < 0.05. All analyses were performed with R software (version 4.4.0) [16].

## Results

**Fig. 1:**
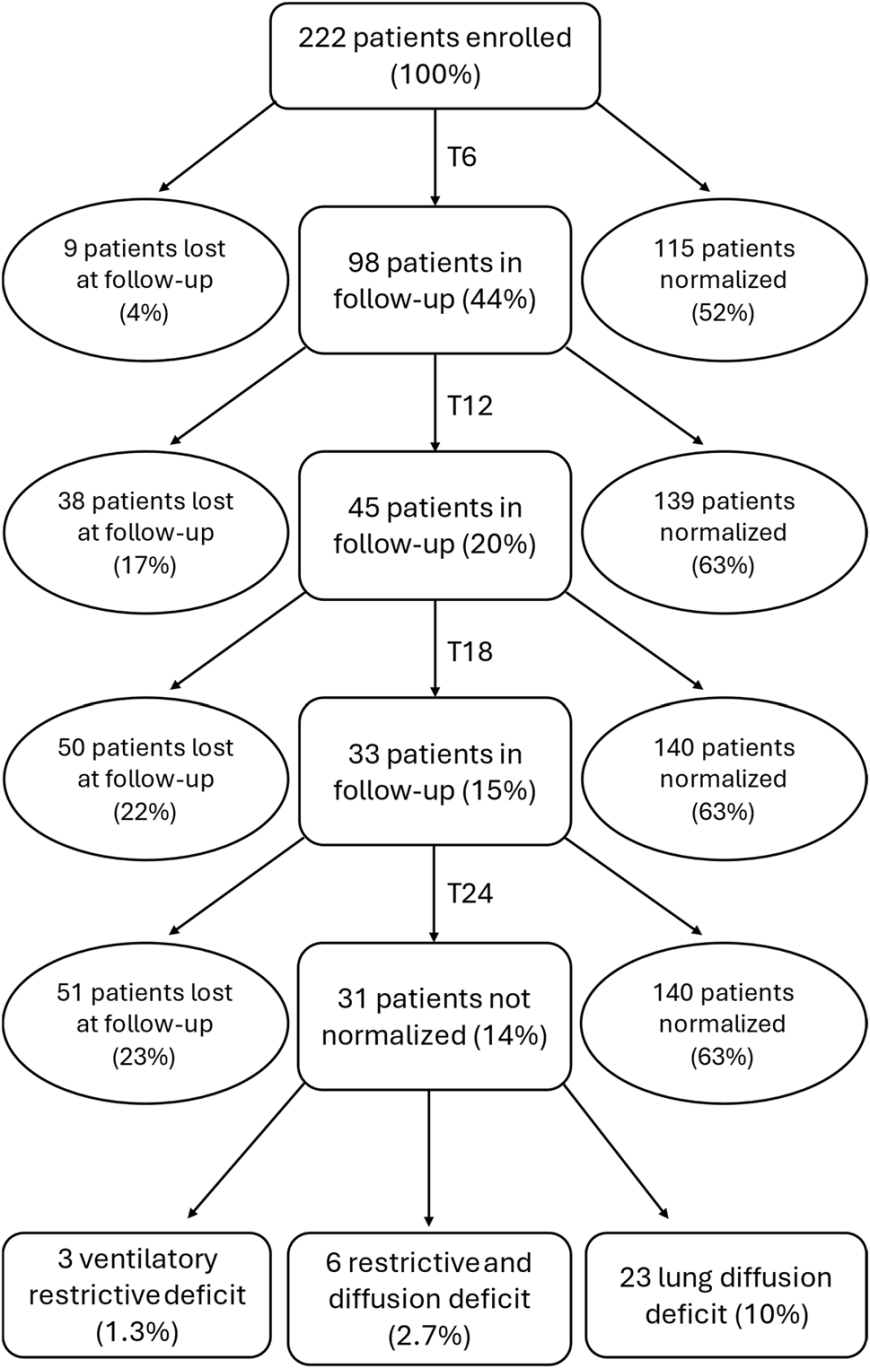
Characterization over time of the patient outcomes under the follow-up cycle at 6, 12, 18, and 24 months

The study was conducted from March 2020 to April 2023. 222 patients discharged from the ICU department of ASST—Spedali Civili di Brescia for severe SARS-CoV-2 pneumonia were enrolled during the study period. Enrolled patients underwent sequential follow-up evaluations at 6, 12, 18, and 24 months. A total of 172 patients (77%) completed the study, while 51 (23%) were lost to follow-up. Among the patients who completed the follow-up series, 140 (63%) achieved normalization of respiratory function and pulmonary diffusion parameters, while 32 (14%) showed persistent ventilatory and/or diffusive deficit (Fig. 1).

The demographics of the population recruited in the study are presented in Table 1. A significantly higher percentage of male subjects (71.62%) than female subjects (28.38%) has been documented (*p* < 0.001). The mean age of the population is 61.3 years, while the mean weight and height are 85.5 kg and 1.68 m, respectively, with a mean BMI of 30.1. The male population has significantly higher mean values for weight and height than females.

**Table 1:** Demographic characteristics of enrolled patients after hospital discharge

	Total	Men	Women	*p*-value
Sex	222	159 (71.62%)	63 (28.38%)	<** 0**.001**
Age	61.34 ± 11.61	61.62 ± 11.12	60.63 ± 12.85	0.569
Weight	85.51 ± 15.45	88.48 ± 14.84	78.03 ± 14.51	<** 0**.001**
Height	1.68 ± 0.10	1.72 ± 0.07	1.58 ± 0.07	<** 0**.001**
BMI	30.12 ± 4.98	29.75 ± 4.79	31.04 ± 5.36	0.083

Data are expressed as mean ± SD

Statistical significance has been established for p < 0.05 (*) and p < 0.001 (**)

**Fig. 2:**
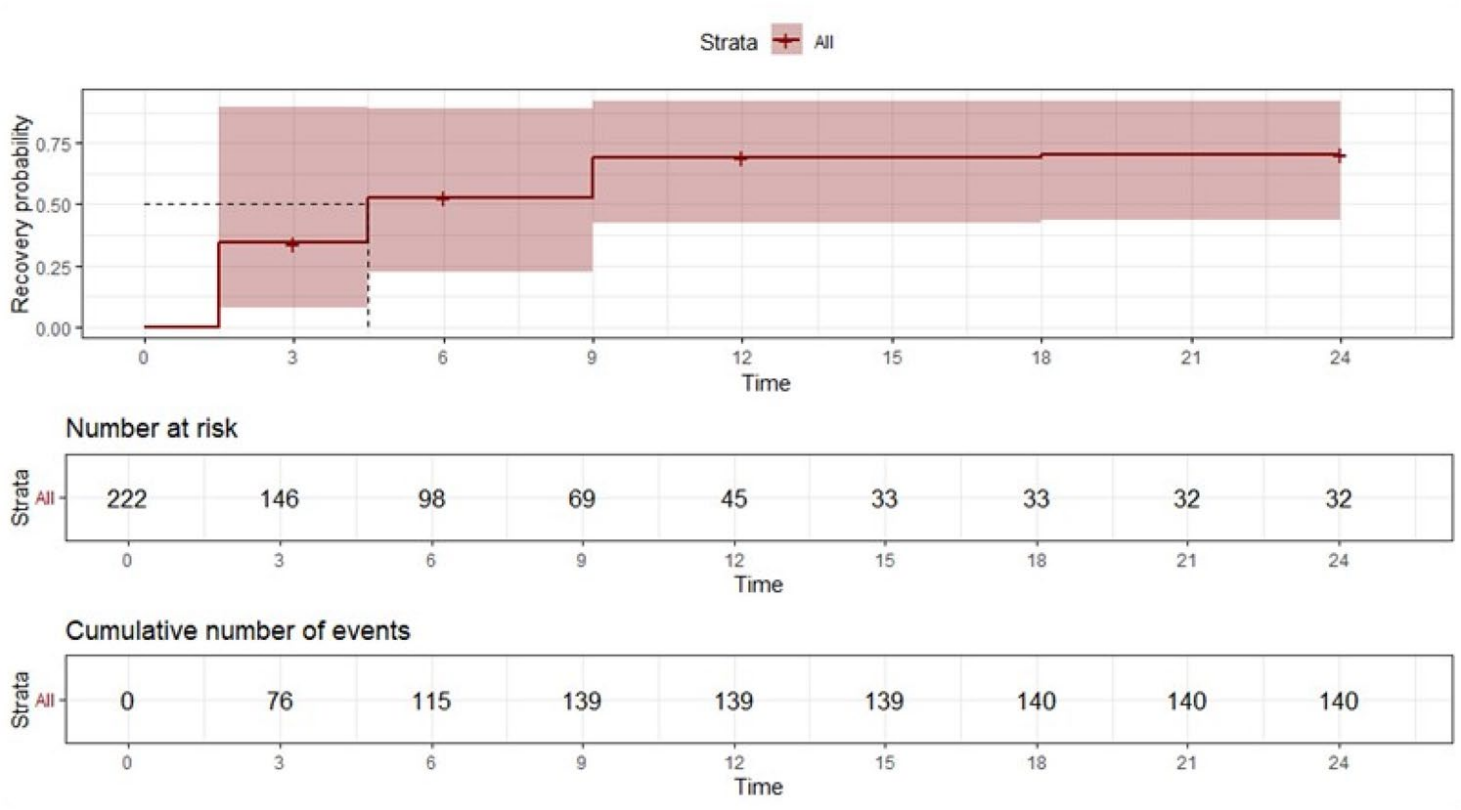
Kaplan–Meier curves for recovery odds analysis with interval-censoring data analysis. The median time to recovery was 4.5 months (95% CI 1.5-NA). The recovery hazard ratio was lower as age increased (HR 0.98, 95% CI 0.97–0.99, *p* = 0.046)

Analyzing the timing of normalization of both ventilatory function parameters and lung diffusing capacity, a median time of 4.5 months (95% CI 1.5-NA) has been documented. This finding is influenced by age as the Hazard Ratio of normalization decreases by 2% as each year increases (HR 0.98, 95% CI 0.97–0.99, *p* = 0.047) (Fig. 2 and Table 2).

**Table 2:** Recovery hazard ratios (HRs) with 95% confidential interval (CI) estimated in the multivariate Cox proportional hazard model

	HR	95% CI	*p*-value
Sex (Ref = male)	1.13	0.76–1.70	0.540
Age (years)	0.98	0.97–0.99	**0**.047*
BMI (Ref = normal)			
Obese	1.37	0.76–2.47	0.301
Overweight	1.19	0.69–2.10	0.534

Statistical significance is established for p < 0.05 (*) and p < 0.001 (**)

The analysis of individual respiratory function parameters revealed a median normalization time of 1.5 months for VC, FVC, FEV_1_, Tiffeneau index, TLC, and KCO regardless of the influence of demographic factors such as gender, age, and BMI, with the only exceptions of the Tiffeneau index in which the hazard ratio indicates a 42.4% reduced odds of normalization in male subjects (HR 0.576, 95% CI 0.388–0.857, *p* = 0.006) and KCO in which the hazard ratio indicates a 116% increased odds of normalization in obese subjects (HR 2.16, 95% CI 1.24–3.77, *p* = 0.007).

The median time to VA normalization was 4.5 months (95% CI 1.5-NA), a result influenced, again, by male sex, in which the Hazard Ratio indicates 40.1% reduced odds of normalization (HR 0.599, 95% CI 0.388–0.857, *p* = 0.006).

Finally, the median time to DLCO recovery was 9.0 months (95% CI 1.5-NA), a result influenced by age and BMI. The hazard ratio indicates a reduced odds of normalization of 3.1% as each year increases (HR 0.969, 95% CI 0.949–0.988, *p* = 0.002) and, in contrast, 226% increased odds of normalization in obese subjects (HR 3.26, 95% CI 0.709–3.14, *p* = 0.003) (Table 3 and Supplemental Material).

**Table 3:** Normalization hazard ratios (HRs) with 95% confidential interval (CI) estimated in the multivariate Cox proportional hazard model for the main respiratory and lung diffusion parameters

	Sex.Male				Age				BMI.Catobese				BMI.Catoverweigth			
	HR	LCI.095	UCI.095	*p*-value	HR	LCI.095	UCI.095	*p*-value	HR	LCI.095	UCI.095	*p*-value	HR	LCI.095	UCI.095	*p*-value
VC	0.835	0.548	1.27	0.399	1	0.988	1.02	0.591	1.33	0.76	2.32	0.32	1.35	0.753	2.42	0.314
FVC	0.818	0.319	2.1	0.677	1.01	0.991	1.02	0.418	1.1	0.623	1.93	0.748	1.16	0.638	2.1	0.63
FEV1	0.847	0.562	1.28	0.427	1.01	0.998	1.03	0.077	1.39	0.541	3.58	0.492	1.23	0.424	3.56	0.704
FEV1/FVC	0.576	0.388	0.857	**0**.006*	0.996	0.982	1.01	0.638	0.962	0.589	1.57	0.878	0.828	0.487	1.41	0.486
RV	0.732	0.412	1.3	0.286	0.988	0.967	1.01	0.264	2.04	0.903	4.61	0.086	1.83	0.812	4.14	0.145
TLC	0.865	0.628	1.19	0.377	0.997	0.981	1.01	0.699	1.49	0.863	2.58	0.152	1.5	0.845	2.66	0.166
VA	0.599	0.414	0.866	**0**.006*	1	0.988	1.02	0.656	1.28	0.696	2.37	0.425	1.3	0.709	2.37	0.399
TLCO	1.24	0.737	2.09	0.419	0.969	0.949	0.988	**0**.002*	3.26	1.51	7.04	**0**.003*	1.49	0.709	3.14	0.292
KCO	1.54	0.991	2.39	0.055	0.984	0.968	1	0.059	2.16	1.24	3.77	**0**.007*	1.39	0.754	2.57	0.291

Statistical significance has been established for p < 0.05 (*) and p < 0.001 (**)

## Discussion

The results characterized the recovery timing of the main respiratory parameters, including lung diffusing capacity, and identified the primary demographic factors that increased or decreased the odds of normalization over time in patients with severe SARS-CoV-2-related pneumonia.

The normalization timing analysis of respiratory function reveals that approximately 14% of the recruited population, 24 months after hospital discharge, still had persistent ventilatory (mainly restrictive) and/or diffusive deficits. Currently, only two studies in the literature have evaluated the sequelae of SARS-CoV-2 infection on respiratory function at least 24 months after hospital discharge, and both corroborate the findings documented in our work, particularly regarding the persistence of the diffusive defect. Faverio et al. conducted a 2-year follow-up and found that several subjects had persistent impairment of the diffusing capacity (DLCO) associated with restrictive lung patterns. Specifically, 49% of the population had DLCO values < 80% pred. [17]. Similarly, the study by Han et al. evaluated lung function and radiological findings in post-COVID-19 hospitalized patients 3 years after discharge. The results indicated that many participants had significant lung function abnormalities, including reduced DLCO and persistent radiological changes, such as ground-glass opacities and reticular patterns on chest imaging [18]. These data underline the chronic nature of lung damage following severe SARS-CoV-2 pneumonia and the need to set up long-term follow-ups.

It is known from the literature that various factors, including age, sex, and obesity, influence the recovery of respiratory function in patients with severe forms of SARS-CoV-2 pneumonia. Each factor significantly determines the recovery time of ventilatory function and lung diffusing capacity [19–22]. Our study demonstrated how the odds of normalization for ventilatory function and diffusing capacity of the lung, particularly DLCO, progressively decrease by 2% for each year of age increase. This finding is consistent with existing literature, which reports that age is a determinant of recovery time after SARS-CoV-2 pneumonia. The elderly population is generally more susceptible to severe respiratory infections and shows prolonged recovery time due to age-related physiological changes, including decreased lung function and altered immune responses. Aging is also associated with increased susceptibility to acute lung injury and chronic pulmonary sequelae after viral pneumonia [23, 24]. Additionally, the elderly population experienced more severe disease manifestations, leading to longer recovery periods [25].

Another factor that can reduce the odds of normalization of the main respiratory function parameters, particularly the alveolar volume (VA), is male gender. Again, the literature confirms what we have documented. Male patients tend to have longer recovery times than females. This disparity may be attributed to differences in immune responses, with women often showing a more robust immune response to viral infections [26]. In addition, the presence of comorbidities, which are more prevalent in the male sex, can further complicate recovery [27]. Finally, it should also be considered how the impact of the immune response is influenced by genetic and hormonal factors [28].

Another demographic factor that can affect the odds of normalization is obesity. Our study documented how obesity increases the odds of normalization of lung diffusing capacity parameters, particularly DLCO and KCO. In this case, what we documented is not unanimously confirmed in the literature. In fact, obese patients often experience slower DLCO recovery than non-obese ones. This phenomenon can be attributed to several obesity-related factors, including increased susceptibility to developing a fibrotic lung pattern, chronic pro-inflammatory status, excessive oxidative stress, impaired immunity, and deregulation of cytokine signaling [29]. Also, several studies identified obesity as a prognostic factor that can increase the risk of developing long-term pulmonary complications that may further delay the DLCO normalization after SARS-CoV-2 infection [30, 31]. In contrast, our findings, which show a faster normalization of respiratory function in relation to obesity, may be explained by the fact that our recruited population, comprising both men and women, had a high basal mean BMI. This issue may have reduced population heterogeneity, thereby influencing our assessments of respiratory function and lung diffusion normalization related to obesity status. Another aspect that needs to be considered is that obese subjects have a higher tendency to develop collateral microcirculation [32]. This could influence alveolar-capillary membrane gas exchanges, which are impaired in subjects with severe forms of SARS-CoV-2 pneumonia due to microthrombi formation, by increasing pulmonary capillary blood volume (Vc), as shown in studies on DLNO/DLCO ratios [33–35]. In addition, it is fundamental to recall that obese subjects, especially those with low comorbidities, have higher baseline DLCO values than non-obese, likely due to greater KCO over time because of increasing Vc [36, 37]. For this reason, the decrease in lung diffusion capacity caused by SARS-CoV-2 pneumonia may be partially mitigated due to constitutively higher DLCO and KCO values. Therefore, obese subjects, although taking longer to return to their baseline lung diffusion values, may still achieve normalized DLCO and KCO values (> 80% pred.) in a shorter amount of time than non-obese subjects. This possibility is further supported by our analysis, which showed that although DLCO required a median of 9 months to normalize, its major determinants (KCO and VA) reached normalization earlier (at 4.5 and 1.5 months, respectively). Therefore, it would be advisable to investigate this aspect further to tailor dedicated clinical recovery protocols for this type of patient. However, this study has some limitations. For instance, chronic respiratory diseases are still underdiagnosed, and in the analysis, we excluded patients with well-documented pre-existing obstructive, restrictive, or mixed ventilatory defects. Therefore, our consecutive recruitment strategy allowed us to focus on the absolute trend of respiratory function parameters during follow-up, regardless of the individual’s baseline clinical profile, and in coherence to provide a real-life depiction in individuals who survived severe SARS-CoV-2 infection. On the other hand, it would have been logistically impractical to retrieve their full clinical histories consistently. Therefore, our study had no control over possible undiagnosed respiratory conditions that may have occurred before hospitalization. Another limitation due to the consecutive recruitment approach of patients, thus allowing to minimize selection bias and reflect the outcomes of patients consecutively admitted for severe SARS-COV2 pneumonia, is that not all data necessary for detailed patient stratification (such as smoking habits and full detailed description of other non-pulmonary comorbidities) could be collected systematically, due to practical constraints such as varying patient availability for extended interviews. However, it is crucial to emphasize that the consecutive recruitment of patients was necessary to provide the most unbiased, real-life trend of respiratory function parameters in patients followed at a clinical level after recovering from a severe COVID-19 exacerbation.

## Conclusions

This study provides valuable insights into the long-term effects of SARS-CoV-2 pneumonia on ventilatory function and lung diffusion capacity. The findings suggest that a significant proportion of patients, particularly those with severe disease, experience persistent ventilatory and diffusive impairments up to 24 months after hospital discharge. Age, sex, and obesity were identified as key demographic factors influencing the odds of normalization of ventilatory function and diffusing capacity of the lung.

While age and male gender were found to decrease the odds of normalization, obesity appears to increase them for parameters such as DLCO and KCO.

To the best of our knowledge, this is one of the few follow-up studies conducted on patients who survived SARS-CoV-2 pneumonia that evaluates a 2-year time window and specifically the only one focusing exclusively on severe forms that required prolonged ICU stays with the need for ventilatory support.

The present findings suggest the importance of long-term follow-up and personalized care for patients with severe SARS-CoV-2 pneumonia, as well as the need for further research to better understand the mechanisms that promote or delay their respiratory function recovery and tailor targeted interventions.

## Supplementary Information

Below is the link to the electronic supplementary material.

Supplementary file1 (DOCX 92 KB)

## Data Availability

The data supporting the findings of this study are available from the corresponding author upon reasonable request.
