# Supplementary material for: 24-Month assessment of respiratory function in patients hospitalized for severe SARS-CoV-2 pneumonia: a follow-up study
Source: Intern Emerg Med. 2025 Nov 3;20(8):2455–62. doi: 10.1007/s11739-025-04153-5 (PMC12672721; doi:10.1007/s11739-025-04153-5)
Supplement: Supplementary file 1 — Supplementary file1 (DOCX 92 KB) [file 11739_2025_4153_MOESM1_ESM.docx]

**VC**

Median time to VC recovery was 1.5 months (95%CI 1.5-1.5).


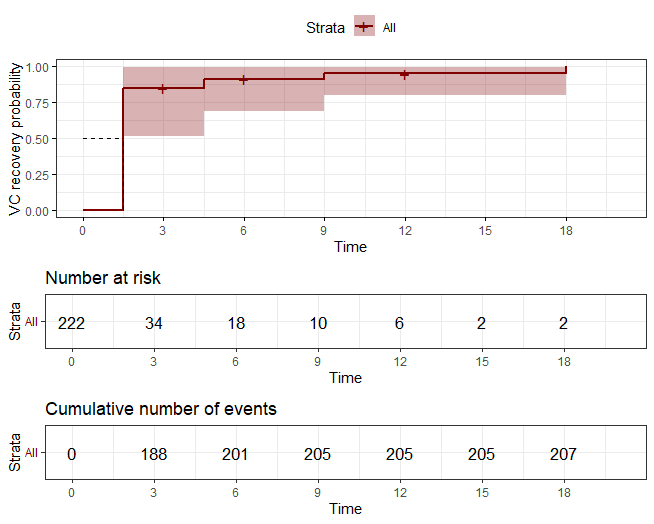


**FVC**

Median time to VC recovery was 1.5 months (95%CI 1.5-4.5).

**
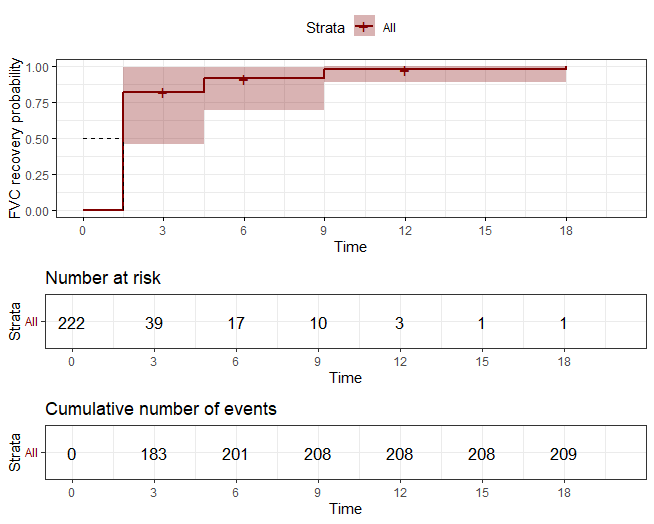
**

**FEV1**

Median time to FEV1 recovery was 1.5 months (95%CI 1.5-4.5).

**
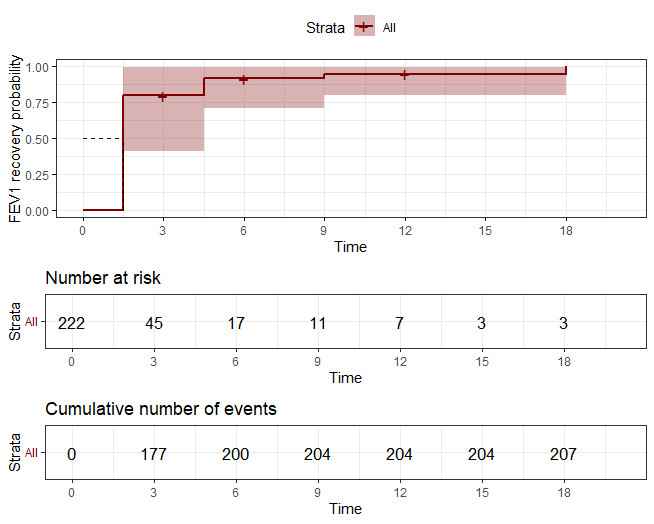
**

**Tiffeneau**

Median time to Tiffeneau recovery was 1.5 months (95%CI 1.5-18.0).

**
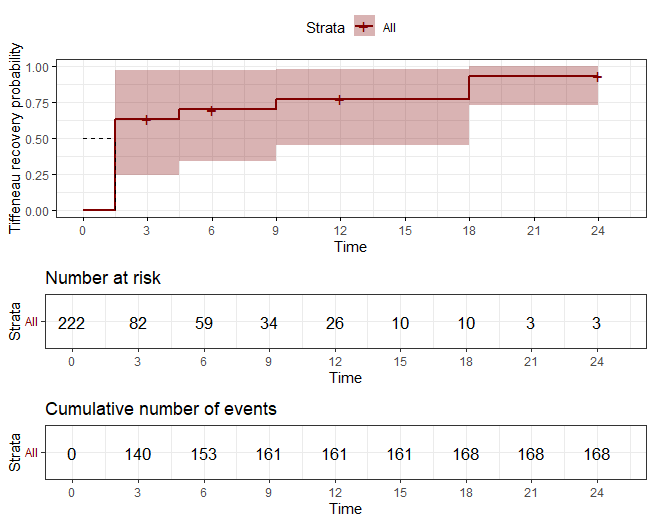
**

**RV**

Median time to RV recovery was not computed since less than half of the sample reached it.

**
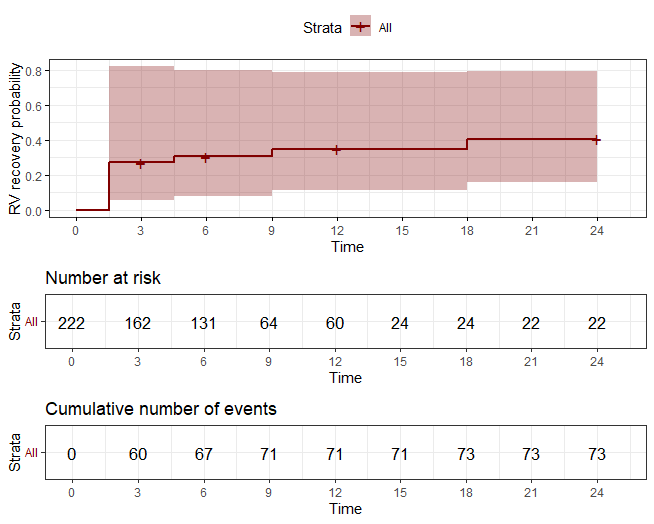
**

**TLC**

Median time to TLC recovery was 1.5 months (95%CI 1.5-9.0).

**
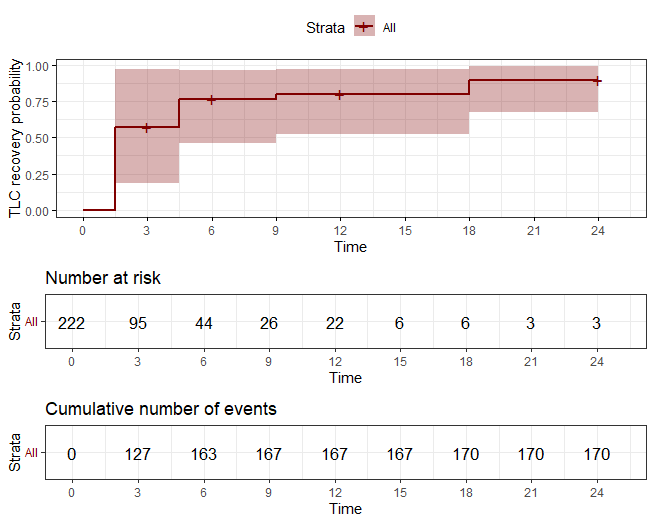
**

**VA**

Median time to VA recovery was 4.5 months (95%CI 1.5-NA).

**
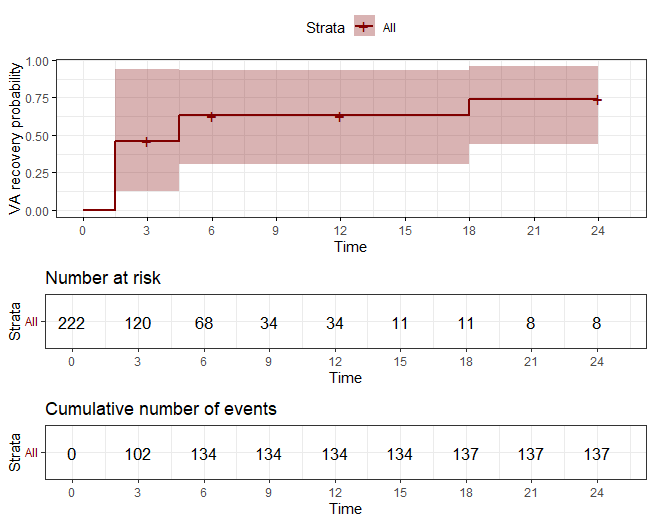
**

**TLCO**

Median time to TLCO recovery was 9.0 months (95%CI 1.5-NA).

**
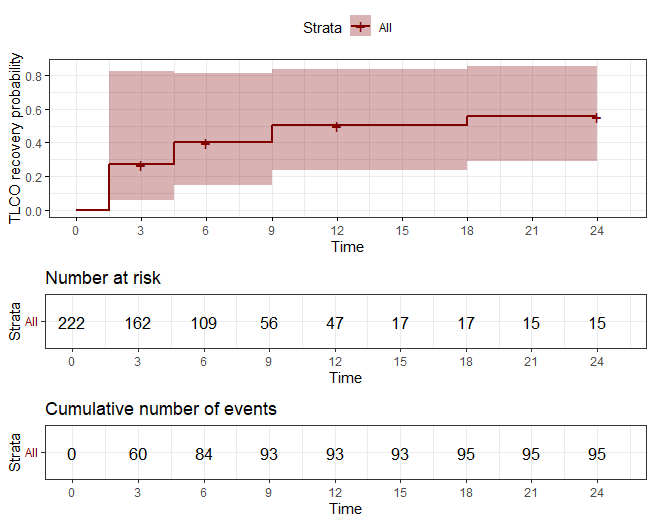
**

**KCO**

Median time to KCO recovery was 1.5 months (95%CI 1.5-9.0).

**
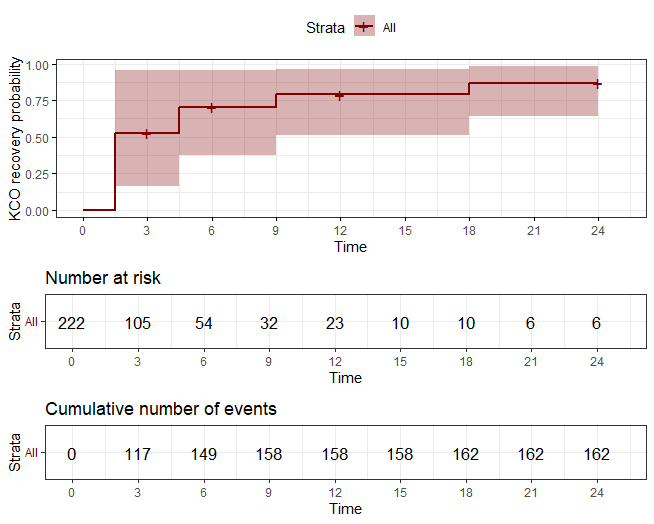
**
